# Principal component analysis of the oxidative stress, inflammation, and dyslipidemia influence in patients with different levels of glucoregulation

**DOI:** 10.5937/jomb0-39636

**Published:** 2023-08-25

**Authors:** Maja Malenica, Aleksandra Klisić, Neven Meseldžić, Tanja Dujić, Tamer Bego, Jelena Kotur-Stevuljević

**Affiliations:** 1 University of Sarajevo, Faculty of Pharmacy, Department of Pharmaceutical Biochemistry and Laboratory Diagnostics, Sarajevo, Bosnia and Herzegovina; 2 University of Montenegro, Faculty of Medicine, Primary Health Care Center, Podgorica, Montenegro; 3 University of Belgrade, Faculty of Pharmacy, Department for Medical Biochemistry, Belgrade

**Keywords:** antioxidants, prooxidants, glycemic control, inflammation, dyslipidemia, antioksidansi, pro-oksidansi, kontrola glikemije, inflamacija, dislipidemija

## Abstract

**Background:**

The aim of the study was to explore the mutual relationship between oxidative stress, inflammation and metabolic biomarkers in subjects with prediabetes (PRE), newly diagnosed type 2 diabetes patients (NT2D) and overt type 2 diabetes (T2D) using principal component analysis (PCA) as a thorough statistical approach.

**Methods:**

Glycated hemoglobin, lipid parameters, inflammation (IL-6, CRP and fibrinogen) and oxidative stress markers pro-oxidants (AOPP, PAB, TOS) and antioxidants (PON1, tSHG, TAS) were measured. PCA was applied to explore the factors that the most strongly influenced glucoregulation.

**Results:**

A total of 278 subjects were (i.e., 37 PRE, 42 NT2D and 99 T2D) were compared with 100 healthy subjects as a control group (CG). PCA emphasized 4 different factors explaining 49% of the variance of the tested parameters: oxidative stress-dyslipidemia related factor (with positive loading of TG and tSHG, and with negative loading of HDL-c and TAS), dyslipidaemia related factor (i.e., total cholesterol and LDL-c, both with positive loading), Anthropometric related factor (i.e., waist and hip circumference, both with positive loading) and oxidative stressInflammation related factor (i.e., PAB, fibrinogen, and CRP all with positive loading). Out of these 4 factors, only oxidative stress - dyslipidaemia related factor showed a significant predictive capability towards poor glucoregulation. An increase in this factor by one unit showed a 1.6 times higher probability for poor glucoregulation.

**Conclusions:**

Redox imbalance (determined with lower TAS and higher tSHG), in addition to higher TG and lower HDLc was associated with poor glucoregulation.

## Abstract (sr)

**Uvod:**

Cilj istraživanja je bio da se ispita povezanost oksidativnog stresa, inflamacije i metaboličkih biomarkera kod pacijenata sa predijabetesom (PRE), de novo dijabetesom (NT2D) i ranije dijagnostikovanim dijabetesom (T2D) pomoću glavne komponentne analize (PCA).

**Metode:**

Glikozilirani hemoglobin, lipidni status, markeri inflamacije (IL-6, CRP i fibrinogen), parametri oksidativnog stresa pro-oksidanti (AOPP, PAB, TOS) i antioksidansi (PON1, tSHG, TAS) su mereni. PCA je primenjena da bi se ispitali faktori koji najviše utiču na glikoregulaciju.

**Rezultati:**

U istraživanje je uključeno 278 ispitanika: 37 PRE, 42 NT2D i 99 T2D, kao i 100 zdravih osoba koji su činili kontrolnu grupu (CG). PCA je izdvojila 4 različita faktora objašnjavajući 49% varijanse ispitivanih parametara: oksidativni stres-dislipidemija faktor (sa pozitivnim uticajem TG i tSHG, i negativnim uticajem HDL-c i TAS), dislipidemija faktor (tj, ukupni holesterol i LDL-c, oba sa pozitivnim uticajem), antropometrijski faktor (tj, obim struka i kukova, oba sa pozitivnim uticajem) i oksidativni stres-inflamacija faktor (tj, PAB, fibrinogen i CRP, svi sa pozitivnim uticajem). Od ova 4 faktora, jedino je oksidativni stres - dislipidemija faktor pokazao značajnu prediktivnu sposobnost za lošu glikoregulaciju. Porast ovog faktora za jednu jedinicu je pokazao 1,6 puta veću verovatnoću za lošu glikoregulaciju.

**Zaključak:**

Redoks disbalans (određen nižim vrednostima TAS i višim vrednostima tSHG), kao dodatak većim vrednostima TG i nižim vrednostima HDL-c su povezane sa lošijom glikoregulacijom.

## Introduction

Oxidative stress is the underlying characteristic of various cardiometabolic disorders [1] [2] [3] [4] [5]. Type 2 diabetes mellitus (T2D) is increasing worldwide, in parallel with the increase of individuals with obesity. It is well known that enlarged visceral adipose tissue is a source of a variety of reactive oxygen/nitrogen species (ROS/RNS) [1] [6] [7]. Besides, it secrets different pro-inflammatory adipokines and cytokines leading to a chronic low-grade inflammation state. As a consequence, insulin signaling pathways become targeted leading to an insulin-resistant state, which affects the liver tissue, adipose tissue and skeletal muscles [8].

Insulin resistance (which is a typical finding in prediabetes and overt diabetes) favours enhancedlipolysis of triglycerides (TG) and causes an increased hepatic flux of free fatty acids (FFA). Moreover, enhanced lipogenesis [i.e., higher concentration of very-low density lipoproteins (VLDL) and small dense low-density lipoproteins (sdLDL)], as well as a reduction in high-density lipoproteins (HDL) and changes in their composition (the state which is described as »atherogenic dyslipidaemia«), is the predictor of atherosclerosis and cardiovascular disease [8] [9].

Prediabetes is an intermediate stage between normoglycemia and overt T2D and is associated witholder age and obesity [10]. Even though that prediabetes is asymptomatic, subjects with pre-diabetes are at risk of progression to T2D, as well as cardiovascular disease [11] [12].

It is of utmost importance to recognize this metabolic disorder as early as possible before overtdiabetes occurs and to delay and/or prevent its further complications, given that clinical symptoms of T2D are often unrecognized.

Hyperglycemia causes increased production of ROS [13]. The moment when antioxidant defensecapacity becomes exhausted signifies the development of a toxic milieu, with concomitant structural and functional changes of deoxyribonucleic acid, proteins, and lipids, which accelerates tissue damage and apoptosis [13].

However, the universal biomarker that best reflects oxidative stress is still unrecognized [14]. In addition, in an attempt to better explain the complex pathophysiological processes and to find the best predictors of cardiometabolic disorders some recent reports included a variety of biomarkers that reflect different signaling pathways of the mentioned disorders. In line with this, we have recently shown that a statistical approach that included principal component analysis (PCA) of various biomarkers, rather than each biomarker alone, can be a better determinant of some metabolic disorders [15] [16] [17]. However, to our knowledge, there are no studies that applied PCA including different oxidative stress, inflammation, and cardiometabolic biomarkers in populations with prediabetes and overt diabetes. Hence, the aim of the current project was to find the best diagnostic approach to poor glucoregulation, which includes a wide spectrum of prooxidants, antioxidants, as well as inflammation, and metabolic biomarkers.

## Materials and methods

### Patients

A total of 278 subjects were included in the study: 37 patients with prediabetes (PRE) (35.2% females), 42 newly diagnosed type 2 diabetic patients (NT2D) (59.5% females), and 99 with type 2 diabetes mellitus (T2D) (52.5% females), who were compared with 100 healthy subjects as a controlgroup (CG) (60.0% females). All participants were consecutively recruited in a period between January 2016 to January 2017 in the Clinical Centre University in Sarajevo and General Hospital Tešanj, Bosnia and Herzegovina. Each examinee completed a questionnaire that was consisted of questions regarding demographic characteristics, lifestyle habits (e.g., cigarette smoking, alcohol use and an open question of somatic illnesses).

The inclusion criteria for participants with PRE and T2D were taken from the 2020 American Diabetes Association Standards of Diabetes Care [18].

Participants were considered to have prediabetes if they were not taking any antihyperglycemic therapy, if they exhibited fasting glucose levels between 5.6 mmol/L and < 7.0 mmol/L, and/or if they had glycated hemoglobin (HbA1c) levels between 5.7% and 6.4%.

The control group consisted of diabetes-free participants who did not use any antihyperglycemic, antihyperlipidemic and antihypertensive medications, with HbA1c levels lower than 5.7% and fasting glucose levels lower than 5.6 mmol/L.

The exclusion criteria were: liver disease (other than steatosis), kidney disease, chronic pancreatitis, gastrointestinal disease, inflammatory bowel disease, endocrine disorders (other than diabetes), infection and using hormonal therapy.

Written informed consent was provided by each examinee. The study was conducted in accordance with the Helsinki Declaration and was approved by the Ethics Committee of Cantonal Hospital Zenica and the International University of Sarajevo.

All patients with T2D used oral antihyperglycemics (metformin and sulfonylureas were used by 86.7% and 43.4 of patients, respectively). There were no participants in the CG, PRE, and NTD2 group who used antihyperglycemic medications, as would be expected based on inclusion/exclusion criteria.

Antihyperlipidemics (i.e., statins) were used by 8%, 19% and 29.3% of participants in the PRE,NT2D and T2D groups, respectively. Anti hypertensive drugs were used by 14%, 30% and 60% of participants in the PRE, NT2D and T2D groups, respectively.

### Methods

Anthropometric measures were obtained from each examinee [i.e., body height and weight and waist and hip circumference (WC and HC, respectively)], whereas body mass index (BMI) and waist-to-hip circumference ratio (WHR) were calculated. Systolic (SBP) and diastolic blood pressure (DBP) were measured with a mercury sphygmomanometer. The measurements were done by the two licenced researchers who were healthcare workers. The measurements were performed following the same protocol.

Blood samples were taken after an overnight fast (of at least 8 hours). HbA1c was measured in the whole blood by the immunoturbidimetry method using the autoanalyzer Dimension Xpand (Siemens, München, Germany). C-reactive protein (CRP) was measured nephelometrically on the same analyzer. Fibrinogen levels were measured on an automatic coagulation analyzer (Sysmex CA-600, Cobe, Japan). For the determination of interleukin-6 (IL-6) flow cytometry was used. For the assay for IL-6 particles with defined fluorescence intensity were used for the detection of soluble cytokine at very low concentrations (10–2500 pg/mL) (Human IL-6 Flex Set, BDTM Cytometric Bead Array).

Oxidative stress status parameters were determined as follows:

Total protein sulfhydryl (tSHG) groups were determined spectrophotometrically using 5, 5’-dithiobis (2-nitrobenzoic acid) [19] [20]. A reaction with potassium iodide and glacial acetic acid was applied for advanced oxidation protein products (AOPP) measurement by the method of Witko-Sarsat et al. [21]. Total oxidative status (TOS) was determined spectrophotometrically using o-dianisidine optimized by Erel [22]. Total antioxidative status (TAS) was obtained spectrophotometrically using ABTS (2,2 -azino-bis-[3-ethyl-benzothiazoline-6-sulfonic acid]) as a chromogen [23].

The measurement of prooxidant-antioxidant balance (PAB) was done using 3,3’, 5,5’-tetramethylbenzidine as a chromogen [24].

### Calculation of scores

The OXY score was calculated by subtracting the protective score (i.e. obtained as an average ofstandardized antioxidant variables (tSHG and TAS, i.e. ANTIOX score) from the damage score (i.e. obtained as the average of standardized prooxidant factors AOPP, TOS, and PAB, i.e. PROOX score) [25].

### Statistical analysis

Statistical analysis was done using SPSS Statistics v.26.0 (IBM Corporation, NY). The normalityof the data distribution was evaluated by the Shapiro-Wilk and Kolmogorov-Smirnov test, where appropriate. Shapiro Wilk test was used to test the normality for the groups <50 participants [i.e.Prediabetic patients (PRE), N = 37 and Newly diagnosed type 2 diabetic patients (NT2D), N = 42)]. Kolmogorov Smirnov test was used to test the normality for the groups > 50 participants [i.e. Type 2 diabetic patients (T2D), N = 99 and Control group (CG), N= 100].

The significance of differences in clinical markers’ concentrations between the groups was estimated using the ANOVA test (for the normally distributed data) and the Kruskal-Wallis test (for the non-normally distributed data). The Tukey’s test was performed for post hoc analysis, as well as the Mann-Whitney test. The results are presented as mean ± standard deviation (SD) and median (25^th^–75^th^ percentile).

Principal component analysis (PCA) [26] with varimax-normalized rotation was used to determineall variables that significantly affected T2D, grouped into several factors. The basic criteria for the variables’ inclusion into the distinct factors were: factor extraction based on eigenvalues higher than 1 and for factor, influence coefficient higher than 0.5. Finally, the number of factors was fixed at 4.

Binary logistic regression analysis was used to test the possible predictive capability of 4 PCA-selected factors which were transformed into 4 separate scores – quantitative variables. The dependent variable in binary logistic regression analysis was glucoregulation status (0-good, 1-poor) and independent variables were 4 new factors created by PCA analysis and presented as their numerical values – scores. Multicollinearity was checked by VIF parameters calculation and all VIFs = 1, which denied multicollinearity between the tested variables. P-values less than 0.05 were considered statistically significant.

## Results

Table 1 shows the descriptive statistics of demographic and anthropometric characteristics of the subjects included in the study. Participants with PRE, NT2D, and T2D were older and displayed higher anthropometric indices (i.e., BMI, WC, HC, WHR), SBP, and DBP vs. CG.

**Table 1:** Demographic and anthropometric characteristics of control (CG), prediabetes (PRE), newly diagnosed Type 2 diabetes mellitus (NT2D), and Type 2 diabetes mellitus (T2D) individuals. † Data are presented as Mean +/- SD (unless otherwise indicated in the «Variables” column) and are compared by one-way ANOVA with the Tukey post hoc test; ^aa^ -P<0.01, ^aaa^0.001 vs. CG<br>CG-Control group; PRE-Prediabetic patients; NT2D-Newly diagnosed type 2 diabetic patients; T2D-Type 2 diabetic patients; BMI-Body mass index; WHR-Waist hip ratio; HC-Hip circumference; WC-Waist circumference; SBP-Systolic blood pressure; DBP-Diastolic blood pressure.

Variables	Control group (CG)<br>N= 100	Patients with<br>prediabetes (PRE)<br>N = 37	Patients with newly<br>diagnosed type 2<br>diabetes (NT2D)<br>N = 42	Patients with type 2<br>diabetes (T2D)<br>N = 99	P
Age, years	48±7	55±11^aaa^	57±7^aaa^	56±6^aaa^	<0.001
Gender<br>Prevalence of<br>females, n (%)	60 (60)	13 (35.2)	25 (59.5)	52 (52.5)	0.081
BMI, kg/m^2^	25.8±3.8	28.9±2.8^aa^	30.7±5.6^aaa^	30.4±4.6^aaa^	<0.001
HC, cm	104.9±8.9	111.2±8.2^aa^	109.0±11.9	110.4±9.9^aaa^	<0.001
WC, cm	91.0±9.4	103.1±9.9^aaa^	103.6±13.1^aaa^	104.1±12.0^aaa^	<0.001
WHR	0.87±0.08	0.93±0.08^aaa^	0.95±0.06^aaa^	0.94±0.07^aaa^	<0.001
SBP, mmHg	120±10	131±12^aa^	137±20^aaa^	136±14^aaa^	<0.001
DBP, mmHg	78.63±7.76	84.55±10.41^aa^	84.63±9.38^aa^	85.20±7.030^aaa^	<0.001
Antihyperglycemics,<br>n (%)	–	–	–	99(100)	/
Metformin	–	–	–	86(86.7)	/
Sulfonylurea	–	–	–	43 (43.4)	/
Antihyperlipidemic<br>agents (statins), n (%)	–	3(8.0)	8(19.0)	29(29.3)	0.026
Antihypertensives, n<br>(%)	–	14 (37.8)	30 (71.4)	60 (60.6)	0.008
Prevalence of<br>smokers, n (%)	32 (32%)	20 (54.0%)	27 (64.3%)	26 (26.3%)	0.761

Also, more individuals with T2D used antihyperlipidemic agents (statins) and antihypertensives, as compared with NTD2 and PRE group.

The levels of clinical markers in the examined groups are shown in Table 2.

**Table 2:** Clinical markers of tested population groups. Data are presented as Mean (range); P – Kruskal-Wallis test; ^aaa^ p<0.001 vs. CG; ^b, bb, bbb^ P<0.05, 0.01, 0.001, respectively vs. PRE; ^c,cc,ccc ^P<0.05, 0.01, 0.001, respectively vs. NT2D.<br>^*^After Bonferroni correction this was not significant any more (P>0.017).<br>CG-Control group; PRE-Prediabetic patients; NT2D-Newly diagnosed type 2 diabetic patients; T2D-Type 2 diabetic patients; HbA1c-Glycated hemoglobin; HDL-c –High-density lipoprotein cholesterol; LDL-c low-density lipoprotein cholesterol; TG-Triglycerides; IL-6-Interleukin 6; CRP-C-reactive protein; UA-Uric acid; AOPP-Advanced oxidation protein products; PAB-Prooxidant-antioxidant balance; TOS-Total oxidant status; PON1-Paraoxonase 1; tSHG-Total sulfhydryl groups; TAS-Total antioxidant status.

Variables	Control group (CG)<br>N=100	Patients with<br>prediabetes (PRE)<br>N = 37	Patients with newly<br>diagnosed type 2<br>diabetes (NT2D)<br>N = 42	Patients with type 2<br>diabetes (T2D)<br>N = 99	P
Glucose, mmol/L	5.0<br>(4.8–5.4)	6.6<br>(6.2–7.0)^aaa^	8.5<br>(7.9–9.3) ^aaa,bbb^	8.1<br>(7.0–10.0) ^aaa,bbb^	<0.001
HbA1c (%)	5.5<br>(5.3–5.7)	6.2<br>(6.1–6.5)^aaa^	7.3<br>(6.9–8.7) ^aaa,bbb^	7.4<br>(6.7–8.6) aaa,bbb	<0.001
Total cholesterol,<br>mmol/L	5.58<br>(4.83–6.45)	5.30<br>(5.00–6.13)	5.85<br>(5.10–6.88)	5.25<br>(4.40–6.00)^a,c^	0.045^*^
HDL-c, mmol/L	1.26<br>(1.03–1.60)	1.22<br>(0.99–1.42)	1.10<br>(0.90–1.45)^a^	1.10<br>(0.96–1.30)^aa^	0.028^*^
LDL-c, mmol/L	3.52<br>(2.89–4.39)	3.22<br>(2.90–4.06)	3.51<br>(2.56–4.63)	2.97<br>(2.15–3.73)^aaa,c^	0.001
TG, mmol/L	1.34<br>(0.99–1.91)	1.92<br>(1.22–2.38)	2.27<br>(1.51–3.25)^aaa^	2.49<br>(1.58–3.45)^aaa,bb^	<0.001
Fibrinogen, g/L	3.20<br>(2.75–3.48)	3.10<br>(2.80–4.10)	3.95<br>(2.93–4.60)^aaa,b^	3.40<br>(3.10–3.90) ^aaa^	<0.001
IL-6, pmol/L	/	1.26<br>(0.80–2.30)	1.81<br>(1.08–3.38) b	1.75<br>(1.23–3.29) ^bb^	0.037^*^
CRP, mg/L	0.22<br>(0.06–0.77)	2.20<br>(0.77–3.20) ^aaa^	3.10<br>(1.50–4.27) ^aaa^	2.70<br>(1.60–4.05) ^aaa^	0.144
UA, μmol/L	231<br>(207–268)	345<br>(293–415)^ aaa^	309<br>(240–370) ^aaa,b^	302<br>(251– 356) ^aaa,bb^	0.020^*^
AOPP, μmol/L	19.1<br>(17.8–22.4)	150<br>(85–188) ^aaa^	150<br>(87–197) ^aaa^	126<br>(80–196) ^aaa^	0.807
PAB, U/L	77.7<br>(70.5–88.2)	80<br>(66–109)	118<br>(103–138) ^aaa,bbb^	130<br>(115–149) ^aaa,bbb,cc^	<0.001
TOS, μmol/L	3.9<br>(3.1–4.4)	142<br>(128–150) ^aaa^	66<br>(23–89) ^aaa,bbb^	130<br>(116–147) ^aaa,ccc^	<0.001
PON1, IU/L	511<br>(202–929)	265<br>(153–532) ^aaa^	276<br>(193–703) ^aaa^	173<br>(125–382) ^aaa,b,ccc^	<0.001
tSHG, mmol/L	0.35<br>(0.32–0.37)	1.29<br>(0.89–1.55) ^aaa^	1.43<br>(1.19–1.62) ^aaa,b^	1.43<br>(1.12–1.64) ^aaa,b^	0.087
TAS, μmol/L	1284<br>(1236–1354)	422<br>(183–593) ^aaa^	299<br>(96–502) ^aaa^	688<br>(367–886) ^aaa,bbb,ccc^	<0.001

Participants with PRE, NT2D and T2D had higher levels of glucose (*p*<0.001), HbA1c (*p*<0.001),TC (*p*=0.045), LDL-c (*p*=0.001) and TG (*p*<0.001), but lower levels of HDL-c (*p*<0.001) compared with CG. Participants with NT2D and T2D had higher levels of IL-6 (*p*=0.037) compared with CG.

Participants with PRE, NT2D and T2D had higher levels of TOS (*p*<0.001), PON1 (*p*<0.001) fibrinogen (*p*<0.001) and uric acid (*p*=0.020) and lower TAS level (*p*<0.001) compared with CG.

**Figure 1:**
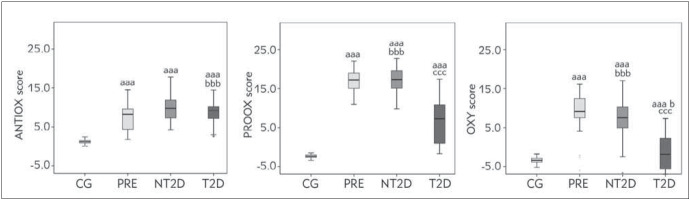
The values of ANTIOX, PROOX and OXY score in examined groups. P- Kruskal-Wallis test; ^aaa ^p<0.001 vs. CG; ^b, bb, bbb^ P<0.05, 0.01, 0.001, respectively vs. PRE; c, cc, ccc P<0.05, 0.01, 0.001, respectively vs. NT2D. CG-Control group; PRE-Prediabetic patients; NT2D-Newly diagnosed type 2 diabetic patients; T2D-Type 2 diabetic patients.

Participants with PRE, NT2D and T2D had higher levels of ANTIOX score (*p*<0.001), PROOX score (*p*<0.001) and OXY score (*p*<0.001), compared with CG (Figure 1).

Principal component analysis was applied to explore the mutual effect of oxidative stress, dyslipidaemia, and inflammation on glucoregulation in studied participants.

PCA was performed in an attempt to derive groups of markers (»factors«) comprising a smaller number of biomarkers that significantly affect glucoregulation.

This analysis identified 4 different factors explaining 49% of the variance of the tested parameters (Table 3). The largest percent of variance (17%) showed oxidative stress-dyslipidemia related factor with positive loadings of TG and tSHG, and with negative loadings of HDL-c and TAS. The second factor explained 11% of the variation and consisted of the dyslipidaemia related factor (i.e., total cholesterol and LDL-c, both with positive loadings). The third factor also explained 11% of the variation and included Anthropometric related factor (i.e., WC and HC, both with positive loadings). The fourth factor explained 10% of the variation and consisted of oxidative stressinflammation related factor (i.e., PAB, fibrinogen and CRP; all with positive loadings).

**Table 3:** Principal component analysis (PCA) derived factors related to T2D. T2D-Type 2 diabetic patients; TG-Triglycerides; tSHG-Total sulfhydryl groups; AOPP-Advanced oxidation protein products; HDL-c-High density lipoprotein cholesterol; TAS-Total antioxidant status; LDL-c-Low-density lipoprotein cholesterol; WC-Waist circumference; HC-Hip circumference; PAB-Pro-oxidant antioxidant balance; CRP-C-reactive protein.

Factors	Included Variables with loadings	Factor<br>variability
Oxidative stress-Dyslipidemia (TG, HDL-c)<br>related factor	TG (0.864)<br>tSHG (0.814)<br>HDL-c (-0.580)<br>TAS (-0.556)	17%
Dyslipidemia (LDL-c) related factor	Total cholesterol (0.919)<br>LDL-c (0.906)	11%
Anthropometric related factor	WC (0.822)<br>HC (0.774)	11%
Oxidative stress-Inflammation<br>related factor	PAB (0.720)<br>Fibrinogen (0.703)<br>CRP (0.650)	10%

We performed binary logistic regression analysis to test the possible predictive capability of 4 PCA-selected factors on glucoregulation. These factors were then transformed into 4 separate scores. Univariate analysis showed the significant predictive capability of factor 1 (oxidative stress – dyslipidaemia (HDL-c) related factor). On the other hand, factor 2 (dyslipidaemia (LDL-c) related factor), factor 3 (anthro pometric related factor), and factor 4 (oxidative stress-inflammation related factor) did not show significant predictive potency towards glycaemic control assessed by HbA1c values. This analysis re vealed that an increase in oxidative stress – dyslipidaemia (TG, HDL-c) related factor (redox disbalance + higher TG and lower HDL-c) by one unit has a 1.6 times higher chance for poor glucoregulation (Table 4).

**Table 4:** Binary logistic regression analysis of poor glycaemic control (HbA1c > 7.0%) predictors. OR- odds ratio; C.I. – confidence interval; # from Omnibus test

Predictors of HbA1c > 7.0%	x^2^, p#	-2 Log<br>likelihood	NegelkerkeR^2^	Precision	OR<br>(95% C.I.)	p
Oxidative stress-Dyslipidemia<br>related factor	4.9, 0.026	88.1	0.076	86.2	1.639 (1.075–2.498)	0.022
Dyslipidemia related factor	12.0, 0.001	81.1	0.178	87.1	0.719 (0.484-1.067)	0.101
Anthropometric related factor	3.1, 0.078	90.0	0.048	86.2	0.777 (0.522–1.158)	0.215
Oxidative stress-Inflammation<br>related factor	2.1, 0.146	91.0	0.033	87.1	0.948 (0.641–1.401)	0.788

## Discussion

As far as we are aware, this is the first research that used PCA of different biomarkers in order toinvestigate complex pathophysiological processes in prediabetes and overt diabetes. Also, this study is unique since it explored a wide spectrum of biomarkers that reflects redox homeostasis (i.e., prooxidants such as PAB, AOPP, TOS and PROOX score and antioxidants such as PON1, TAS, tSHG and ANTIOX score, as well as its comprehensive OXY score) and inflammation status (CRP, IL-6, fibrinogen) in diabetes. To better enlighten the mentioned processes we included participants with different glucoregulation status such as prediabetes and NT2D (both groups without antihyperglycemic medication use) and T2D, compared with the healthy control group.

As it would be expected, the control group of healthy participants displayed significantly lower levels of oxidative stress and inflammation as reflected by the levels of AOPP, PAB, TOS (Table 2), ANTIOX score, PROOX score and OXY score (Figure 1), and the higher level of PON1 and TAS (Table 2) as compared with NT2D and T2D.

Fluctuations in glucose level are related to oxidative stress [13]. ROS production is directly induced by the severity and the length of hyperglycemia. ROS affect multiple signalling pathways (e.g., activation of the protein kinase C isoform, higher hexosamine pathway flow and higher polyols formation) [13].

A significant difference in tSHG values (which represents the major part of antioxidants in the human body) was observed in PRE, NT2D and T2D, respectively, compared to controls (Table 2).Significantly lower tSHG levels were also observed in PRE compared with NT2D and T2D patients.

This may be explained by increased antioxidative defense capacity when hyperglycemia occurs in an attempt to cope with the increased generation of ROS [1] [6]. Our results are in line with a previous study [27] that reported higher tSHG levels (i.e. total thiols) in patients with diabetes mellitus, as well as in patients with pre-diabetes, when compared with a control group. This suggests that a higher level of antioxidants may represent a compensatory mechanism for the increased production of ROS due to prolonged hyperglycemia. One of the possible explanations related to higher levels of several prooxidants and lower levels of several antioxidants in NT2D as compared to T2D may lie in the fact that participants with T2D used antihyperglycemic and antihyperlipidemic medications that might diminish oxidative stress and inflammation [28]. Moreover, the possibility that some of them may also have used antioxidant supplements which might modulate the level of oxidative stress, cannot be ruled out [29]. However, based on the results of the current study, compared with healthy counterparts, participants with prediabetes had higher levels of prooxidants (i.e., AOPP, TOS, PROOX score, and OXY score) and lower levels of antioxidants (i.e., PON1, and TAS) (Table 2, Figure 1). This is supported by the notion that hyperglycemia even in prediabetes is reflected by increased production of ROS and concomitant insulin resistance is tightly connected with increased oxidative stress [8]. Prolonged hyperglycemia leads to depletion of antioxidant enzymes activity, favoring the increase in ROS, prooxidants and overt diabetes [1] [6] [8]. Once the diagnosis of T2D is confirmed, antihyperglycemic medications that patients use may further modulate/decrease the level of oxidative stress [30]. This might in part explain the results obtained in the current study. Also, our results might support the notion that timely diagnosis of prediabetes is of great importance and use of antihyperglycemic medications in prediabetes might reduce oxidative stress and prevent overt T2D.

Binary logistic regression analysis showed significant predictive capability of only oxidative stress –dyslipidaemia (HDL-c) related factor, whereas the other factors did not show significant predictive potency towards glycaemic control assessed by HbA1c values. An increase in oxidative stress – dyslipidaemia (HDL-c) related factor (i.e., redox disbalance + higher TG and lower HDL-c) showed a 1.6 times higher possibility for poor glycaemic control (Table 4). In a previous study [5], we have shown that the comprehensive DOI score (that represented the summary involvement of dyslipidemia, oxidative stress and inflammation) is an independent predictor of HbA1c level, although the study did not include newly diagnosed patients with T2D and despite the fact that all studied patients with T2D used antihyperglycemic medications. In this study we showed that as the DOI score rose by 1 unit, the probability for higher HbA1c levels increased by 9%. This appears to confirm the complex relationship between all of these pathophysiological processes.

As previously stated, visceral adipose tissue (in routine practice most often determined by WC) secretes proinflammatory adipokines and cytokines which are shown to have an adverse impact on insulin signalling pathways [1] [6]. Indeed, we have shown higher levels of IL-6 and fibrinogen in NT2D and T2D compared to prediabetes, as well as higher levels of CRP in prediabetes, NT2D and T2D compared to healthy controls. In parallel with the extent and duration of obesity, insulin resistance favours the increase in lipolysis of TG and consequently the increase of free fatty acids (FFA) secretion from adipose tissue. These processes precede oxidative phosphorylation, peroxidation of lipoproteins in the liver and free radical production [1]. The increased hepatic production of VLDL which are enriched with TG and are assumed to be very lipotoxic are the consequences of an insulin-resistant state, also. The transformation of HDL particles into the smaller HDL3 particles and the increased production of small dense LDL [9], both of which are shown to be prone to oxidative modifications, is another consequence of the insulin-resistant state, thus enabling the beginning and the progression of atherosclerotic alterations [30].

Taking into account all these complex processes and different pathways of cardiometabolic consequences of the insulin resistance state, we aimed to find the best diagnostic approach for the mentioned disorder. The different level of glucoregulation, i.e. insulin-resistant state in subgroups of examined participants, is one of the strengths of the current study since we included participants with prediabetes, NT2D (both groups without antidiabetic therapy), and T2D (on antidiabetic therapy). Also, as previously stated, a variety of redox homeostasis and inflammation biomarkers were explored, as well as factorial analysis/PCA as a reliable statistical approach to investigate the joint effect of oxidative stress, dyslipidaemia, and inflammation on T2D.

The limitations of this study include its crosssectional design, which restricts our ability to definecausality between examined variables. Although a pattern of non-specific marker levels can result in a «score” that can be more specific than the individual marker, it is worth mentioning that the data used in PCA represent a collection of more or less non-specific markers, that can be increased due to more than one mechanism, which suggests that the PCA analysis may not always be reliable. Moreover, there is a possibility that some of the participants may also have used antioxidant supplements, which might modulate the level of oxidative stress and this cannot be ruled out. Other limitations include the relatively small number of participants included in the study. Also, due to the lack of diversity within the study groups the data cannot be readily applied to non-Caucasians. Longitudinal studies with different ethnic groups are needed to further elucidate this issue.

## Conclusions

As far as we know, this is the first study that used factorial analysis of different factors consisted of cardiometabolic biomarkers, markers of oxidative stress and inflammation in participants with different levels of glucoregulation. In line with this, we have shown that oxidative stress – dyslipidaemia (HDL-c) related factor showed a significant predictive capability towards poor glycaemic control. An increase in oxidative stress – dyslipidaemia (HDL-c) related factor (i.e., redox imbalance, in addition to higher TG and lower HDL-c) by one unit showed a 1.6 times higher probability for poor glucoregulation.

## Dodatak

### Conflict of interest statement

All the authors declare that they have no conflict of interest in this work.
